# Development and validation of an occurrence-based healthy dietary diversity (ORCHID) score easy to operationalise in dietary prevention interventions in older adults: a French study

**DOI:** 10.1017/S0007114523002520

**Published:** 2024-03-28

**Authors:** Anne-Fleur Jacquemot, Rosalie Prat, Rozenn Gazan, Christophe Dubois, Nicole Darmon, Catherine Feart, Eric O. Verger

**Affiliations:** 1 Université de Bordeaux, INSERM, BPH, UMR1219, F-33000 Bordeaux, France; 2 ORS PACA, Faculté des Sciences Médicales et Paramédicales, Observatoire Régional de la Santé Provence–Alpes–Côte d’Azur, Marseille, 13385, France; 3 MS-Nutrition, Marseille, France; 4 Trophis, 13170 Les Pennes Mirabeau, France; 5 MoISA, Université de Montpellier, CIRAD, CIHEAM-IAMM, INRAE, Institut Agro, IRD, Montpellier, France

**Keywords:** Healthy dietary diversity, Older adults, Interventional research, Consumption occurrence

## Abstract

Healthy diet and dietary diversity have been associated with healthy ageing. Several scores have been developed to assess dietary diversity or healthy diets in epidemiological studies, but they are not adapted to be used in the context of preventive nutrition interventions. This study aimed to develop an occurrence-based healthy dietary diversity (ORCHID) score easy to implement in the field and to validate it using dietary data from older participants in the latest French food consumption survey (INCA3). The ORCHID score was made of several components representing the consumption occurrences of twenty food groups, in line with French dietary guidelines. The score was then validated using dietary data (namely three 24-h recalls and a food propensity questionnaire) from 696 participants aged 60 years and over in the INCA3 survey. Score validity was evaluated by describing the association of the score with its components, as well as with energy intakes, solid energy density (SED) and the probability of adequate nutrient intakes (assessed by the PANDiet). Higher scores were associated with more points in healthy components such as ‘fruits’ and ‘vegetables’ (*r* = 0·51, and *r* = 0·54, respectively). The score was positively associated with the PANDiet (*r* = 0·43) and inversely associated with SED (*r* = −0·37), while no significant association was found with energy intakes. The ORCHID score was validated as a good proxy of the nutritional quality of French older adults’ diets. It could therefore be a useful tool for both public health research and nutrition interventions.

Increased longevity, lower fertility rates and progress in medicine and pharmacology are contributing to the ageing of the world’s population. In France, the current share of the population aged 65 years and over is approximately 20·5 %^(1)^ and is expected to steadily increase up to 30 % in 2050^(2)^. With this rise, age-related chronic illnesses increase proportionately^(3)^. Healthy dietary patterns throughout life may delay the onset of late-life chronic diseases^(4)^ and many healthy diet scores exist^(5,6)^ but not especially for older people. The ability to maintain such patterns appears even more critical during later life stages, when socio-demographic changes associated with ageing (e.g. widowhood, clinical disorders, poor oral health, etc.) may lead to a loss of appetite and the risk of undernutrition^(7,8)^. Diversity is one sub-constructs of healthy diet according to Seligman’s paradigm^(9)^. Dietary diversity (typically defined as the number of different foods or food groups consumed over a given period) may help postpone the onset of age-related chronic diseases. For instance, longitudinal studies exploring links between dietary diversity and health for older adults report that greater dietary diversity is associated with a reduced risk of all-cause mortality^(10,11)^, frailty^(12)^ and cognitive decline^(13)^. Furthermore, dietary diversity is promoted in most Western countries’ dietary guidelines for the general population^(14,15)^.

Dietary diversity in the general population is associated with nutritional quality^(16)^, assessed by the adequacy of nutrient intakes with nutrient-based recommendations. Nevertheless, a major dimension of diet quality, moderation – defined as the avoidance or limited consumption of nutrients, foods or food groups considered to be unhealthy when consumed in excess – is rarely considered by existing indicators of dietary diversity^(15)^. To overcome this pitfall, certain authors have proposed the concept of healthy dietary diversity, that is, balanced diversity, with a high consumption of a variety of healthful, nutrient-dense foods and a low consumption of energy-dense, nutrient-poor and less healthful foods based on dietary guideline of the country^(17,18)^. Some scores have been developed and validated by evaluating their content and construct validity for the general population^(17,18)^. To calculate these healthy diversity scores as for other type of healthy diet scores^(5,6)^, precise data, including weighted food portions, are needed. No measure of healthy dietary diversity or healthy diet that can be easily operationalised in intervention studies has been proposed, especially for older adults. The aims of this study are to develop an occurrence-based healthy dietary diversity (ORCHID) score easy to implement in the field and to validate it using dietary data from older participants in the latest French food consumption survey (INCA3).

## Methods

### Principle of the ORCHID score

To obtain a tool for measuring dietary diversity that could be easily used in nutritional prevention interventions, we chose to develop a score based on the occurrence of consumption of certain food groups, which would not require information on the weight of the food consumed. In addition, we aimed to develop a score that reflected both the concept of healthy dietary diversity (i.e. diet adequacy and moderation) in which the whole diet is considered^(18)^ and the 2021 French dietary guidelines for older adults^(19)^, with an emphasis on protein-rich foods due to the importance of adequate protein intake for healthy ageing^(19)^.

Essentially, the ORCHID score involves twenty ORCHID components corresponding to twenty ORCHID food groups.

Because the ORCHID score was created for older adults, an emphasis on rich proteins food was made. To create the twenty ORCHID food groups, some food groups rich in proteins from 2021 French dietary guidelines had been reshaped: the food group ‘poultry-fish-eggs-meat’ has been modified into ‘fatty fish’, ‘lean fish and shellfish’, ‘poultry’, ‘eggs’ and ‘meat excluding poultry’; ‘deli meat’ has been modified into ‘cooked ham’ and ‘deli meat excluding cooked ham’ and ‘dairy products’ have been modified into ‘cheese’ and ‘milk and fresh dairy product’. Moreover, because of the importance of pleasure and the risk of energy malnutrition in older adults^(20)^, energy-dense food groups that the 2021 French dietary guidelines advise to avoid excessive consumption (i.e. energy-dense food groups such as ‘sweetened products’ and ‘salted aperitif product’) have been considered positively until a threshold decided with a group of experts.

Each ORCHID component corresponds to the total number of points calculated for each consumption occurrence of the corresponding ORCHID food group. If the 2021 French dietary guidelines advice favouring the consumption of a food group, then a positive rating is used:

ORCHID component = (Number of occurrence) * weight

If the French dietary guidelines advice limiting the consumption of a food group, then a threshold rating is used:

- If number of occurrences =< threshold, ORCHID component = (Number of occurrences)
- If number of occurrences > threshold, ORCHID component = (Number of occurrences - threshold) * weight

The thresholds and the weights were decided based on the expert judgement.

Then, the twenty ORCHID components are summed up to obtain the final ORCHID score.

Among the twenty ORCHID components, thirteen are related to food groups known to be consumed on a daily basis in the French population (e.g. starches and potatoes) and/or that have a daily consumption recommendation in the 2021 French dietary guidelines^(19)^ (e.g. the recommendation to eat five portions of vegetables every day). Those thirteen ORCHID food groups are the following: ‘meat excluding poultry’; ‘cooked ham’; ‘deli meats excluding cooked ham’; ‘milk and fresh dairy products’; ‘cheese’; ‘refined starches and potatoes’; ‘vegetables’; ‘fruits’; ‘oils’; ‘butter, margarine and fresh cream’; ‘salted aperitif products’, ‘sweetened products’ and ‘sweetened beverages’.

The remaining seven ORCHID components are related to food groups known to be infrequently consumed by the French population (e.g. wholemeal products) and/or with a weekly consumption recommendation (e.g. the recommendation to eat fatty fish once a week). Those seven ORCHID food groups are the following: ‘poultry’; ‘eggs’; ‘fatty fish’; ‘lean fish and shellfish’; ‘legumes’; ‘nuts’ and ‘wholemeal or semi-wholemeal products’.

### Population sample and dietary data

To test the validity of the ORCHID, we made secondary analyses on data from the Third French Individual and National Food Consumption Survey 2014–2015 (INCA3) led by ANSES. INCA3 is a cross-sectional survey aimed at estimating the food consumption and eating habits of individuals living in France. The study was carried out between February 2014 and September 2015 among a representative sample of individuals living in mainland France (excluding Corsica and overseas French territories). A total of 3157 eighteen- to seventy-nine--year-old adults participated in the study.

Individuals were selected according to a three-stage cluster sampling design (geographical units, households and individuals), based on the 2011 annual national census, with geographical stratification (region, size of urban area) to ensure national representativeness.

The study sample included participants 60 years of age and over (*n* 1008). Older adults who responded to less than three 24-h dietary recalls or who failed to respond to the self-administered food propensity questionnaire (FPQ) were excluded from the analyses, leading to a sample of 696 older adults (293 men and 403 women).

Participants received a home visit to take anthropometric measures and explain how and when to complete the self-questionnaire. Dietary intakes were then assessed using a series of three non-consecutive 24-h dietary recalls (two on weekdays and one during the weekend) over the 3 weeks after the home visit. During phone interviews conducted by professional investigators, participants were asked to declare all foods and beverages they had consumed the day prior to the call. Every declaration was computerised using GloboDiet (previously known as EPIC-Soft^(17)^) by professional interviewers specifically trained in the methods and the software used. Foods included both simple foods (e.g. apples, meat, etc.) and complex dishes with multiple ingredients (e.g. apple pie, lasagne, meat and vegetable stew, etc.). Portion sizes were estimated by participants during the call, using validated photographs from a picture book of food portion sizes and household measures^(16)^. The nutrient contents of the foods and beverages cited by participants were extracted from the 2016 food composition database of the French Information Centre on Food Quality^(18)^. Participants were also asked to complete the FPQ describing their usual consumption frequency of approximately sixty foods or food groups over 12 months. FPQ are qualitative FFQ that may be used alongside 24-h dietary recalls to estimate long-term food intake. The FPQ was completed either on paper or directly online. Daily nutritional intakes were estimated (without taking into consideration alcohol consumption and without excluding the consumption of food supplements) for each individual over the three 24-h dietary recalls.

### Calculation of ORCHID score in INCA3

#### Calculation of the thirteen ORCHID components based on data from the 24-h dietary recalls

Each food associated with the 24-h dietary recalls was categorised into one of the thirteen ORCHID food groups. Each food declaration was considered only if its ORCHID food group’s daily consumption was more than half of a standard portion (in g) as defined in the French Nutrition and Health Survey (ENNS) and the French ‘Market Research Group for Collective Catering and Nutrition’ (GEMRCN)^(21)^ (Table 1). For example, if less than 25 g of ‘cooked ham’ per day is declared whereas the standard portion size is 50 g, then the ‘cooked ham’ consumption is not taken into account.

**Table 1.** ORCHID food group, standard portions and scoring

ORCHID food group	Standard portion size	Method of calculation of occurrences	Scoring	2021 French dietary guidelines^(19)^
Poultry (and rabbits)	(100 g) ENNS*	Weekly frequency	OC‡ = number of occurrences*2	Eat at least once a day, choosing from poultry, fish, eggs or meat
Meat excluding poultry	(100 g) ENNS	Total number of occurrences on three 24 h recalls	• If number of occurrences =< 1·5, OC = number of occurrences • If number of occurrences > 1·5, OC = (number of occurrences – 1·5)*(-1)	Eat at least once a day, choosing from poultry, fish, eggs or meat For high consumers of meat other than poultry, there is no nutritional benefit in eating it systematically at every meal
Cooked ham	(50 g) GEMRCN†	Total number of occurrences on three 24 h recalls	• If number of occurrences =< 1·5, OC= number of occurrences • If number of occurrences > 1·5, OC= (number of occurrences – 1·5)*(-1)	Avoid consuming it every day and avoid excessive consumption
Deli meat excluding cooked ham	(50 g) GEMRCN	Total number of occurrences on three 24 h recalls	• If number of occurrences =< 1·5, OC = number of occurrences • If number of occurrences > 1·5, OC = (number of occurrence – 1·5)*(-2)	Avoid consuming it every day and avoid excessive consumption
Eggs	(100 g) ENNS	Weekly frequency	OC = number of occurrences	Eat at least once a day, choosing from poultry, fish, eggs or meat
Fatty fish	(100 g) ENNS	Weekly frequency	OC = number of occurrences*2	For large consumers of fish, vary the species and places of supply to limit exposure to environmental contaminants
Lean fish and shellfish	Fish (100 g) ENNS. Shellfish (75 g) Canada	Weekly frequency	OC = number of occurrences*2	For large consumers of fish, vary the species and places of supply to limit exposure to environmental contaminants
Milk and fresh milk dairy	Dairy products (125 g) ENNS. Milk (150 g) ENNS	Total number of occurrences on three 24 h recalls	OC = number of occurrences	2–3 dairy products a day
Cheese	(30 g) ENNS	Total number of occurrences on three 24 h recalls	OC = number of occurrences	2–3 dairy products a day
Legumes	(200 g) ENNS	Weekly frequency	OC = number of occurrences*2	At least 2 times/week
Nuts	(15 g) USA	Weekly frequency	OC = number of occurrences*2	A handful per day

ORCHID food group	Standard portion size	Method of calculation of occurrences	Scoring	2021 French dietary guidelines^(19)^
Refined starches (including bread) and potatoes	Cereals (200 g) ENNS. Bread 50 g ENNS. Potatoes 150 g ENNS	Total number of occurrences on three 24 h recalls	OC = number of occurrences	To be eaten every day, with a preference for wholemeal or slightly refined products over refined products
Semi- or wholemeal products (including bread)	Cereals 175 g) ENNS. Bread 50 g ENNS	Weekly frequency	OC = number of occurrences*2	To be eaten every day, with a preference for wholemeal or slightly refined products over refined products
Vegetables	Vegetable (80 g) ENNS. Soup 250 g AIIBP-FAIBP	Total number of occurrences on three 24 h recalls	OC = number of occurrences*2	At least five portions of vegetables or fruits/d
Fruits	Fruits (80 g) ENNS. Dried fruits 40 g Canada	Total number of occurrences on three 24 h recalls	OC = number of occurrences*2	At least five portions of vegetables or fruits/d
Oils	(8 g) GEMRCN	Total number of oils on three 24 h recalls	OC = ∑ different type of oil + ∑ (different mixed oil)*2	Favour colza, nut and olive oil. Other added fat can be consumed every day in small quantities
Butter, margarine, fresh cream	Butter (8 g). Fresh cream (8 g), GEMRCN	Total number of occurrences on three 24 h recalls	• If number of occurrences =<3, OC= number of occurrences • If number of occurrences > 3, OC = (number of occurrences - 3)*(-2)	Favour colza, nut and olive oil. Other added fat can be consumed every day in small quantities
Salted aperitif products	(30 g) GEMRCN	Total number of occurrences on three 24 h recalls	• If number of occurrences =<3, OC= number of occurrences • If number of occurrences > 3, OC = (number of occurrences – 3)*(-2)	Avoid excessive salt consumption
Sweetened products (including sugar)	Sugar (5 g), dairy desserts (100 g) GEMRCN, biscuits (30 g) ENNS, cereals for breakfast (30 g) ENNS, candies, chocolate, etc. (30 g) GEMRCN	Total number of occurrences on three 24 h recalls	• If number of occurrences =<9, OC= number of occurrences • If number of occurrences > 9, OC = (number of occurrences – 9)*(-2)	Avoid excessive consumption of sweet products
Sweetened drinks (including juice)	(200 g) GEMRCN	Total number of occurrences on three 24 h recalls	• If number of occurrences =<3, OC = number of occurrences • If number of occurrences > 3, OC = (number of occurrences – 3)*(-2)	Avoid excessive consumption of sweet products

* The French Nutrition and Health Survey (ENNS).

† French market research group for collective catering and nutrition (GEMRCN).

‡ ORCHID component.

Food taken into account consisted of both simple foods and complex dishes. The categorisation of simple foods into ORCHID food groups was based on the existing INCA3 food categorisation. About 70 % of the foods were successfully classified among the thirteen ORCHID food groups. The remaining foods were not included in the ORCHID calculation (mostly water, spices, herbs and sauces). Approximately 5 % of the data collected over the three non-consecutive 24-h dietary recalls concerned complex dishes.

Complex dishes were disaggregated into ingredients using recipes, and the main ingredients (i.e. those whose sum represented half of the recipe) were identified. Only the main ingredients were sorted into one of the ORCHID food group and each main ingredient was considered as one occurrence only if the quantity consumed of the ORCHID food group represent more than half a standard portion.

For each individual and each of the thirteen ORCHID food groups, the ORCHID components are calculated from the total number of consumption occurrences in three 24 recalls. The details of the scorings are fully described in Table 1 and had been decided based on expert judgements.

#### Calculation of consumption occurrences based on data from the food propensity questionnaire

For the remaining seven ORCHID food groups, the associated ORCHID components are based on data from the FPQ. To overcome missing occurrences due to a lack of response in the FPQ, an imputation was computed using the random forest method^(22)^, taking into consideration individual characteristics, INCA3, food group quantities and FPQ frequencies with no missing values. To maintain consistency with weekly dietary recommendations, the monthly consumption frequencies collected by the FPQ were transformed into weekly frequencies.

For each individual and each of the seven ORCHID food groups, the total number of consumption occurrences (based on weekly frequencies) is used to calculate the ORCHID components, using a positive, proportional rating system, as French dietary guidelines advice favouring the consumption of foods in these food groups (Table 1).

An example of an ORCHID score’s individual calculation is presented in online Supplementary Material 1.

A toy data set created from the INCA3 data and the R code are available as online Supplementary Materials 2, 3 and 4.

#### Evaluation of ORCHID score validity

To evaluate the content and construct validity of the score, we applied a strategy commonly used for diet quality scores^(5,17,21)^ summarised by Bland and Altam^(22)^. Content validity is met if the score seems complete (i.e. it does not lack any important component), and construct validity is met if the score is associated with the same variables than the object it measures, that is, diet quality. First, content validity was evaluated by (i) examining the distribution of the ORCHID score and its components throughout the sample and (ii) examining the correlation between the ORCHID score and its components. Examining the correlations of each of the components with the total score is important to understand how the score works and what it could imply for its interpretation in various research applications^(6)^. Second, the construct validity of the ORCHID score was evaluated by (i) examining the correlation between the score and daily consumption (in g/d) of the twenty ORCHID food groups, based on the hypothesis that participants with a higher ORCHID score would more likely consume more fruits and vegetables and eat fewer products with a high fat, salt and sugar content than participants with an ORCHID lower score, (ii) examining the correlation between the energy, total food and nutrient intakes, as well as diet quality measures, based on the hypothesis that participants with a higher score would likely have a more nutritionally adequate diet and (iii) examining the relation with socio-demographic and anthropometric variables known to correlate with diet quality: educational level^(23)^, sex^(5)^, BMI^(5,23,24)^, monthly household income^(25)^, number of persons living in the household^(25)^, smoking status^(26)^ and physical activity level^(5)^.

Retained diet quality measures consisted of the solid energy density (SED), a proxy of low nutritional quality, the mean adequacy ratio (MAR), a measure of nutritional adequacy and the PANDiet, a measure of overall nutritional quality, on individual diets. These diet quality measures (SED, MAR, PANDiet) were estimated from the daily nutritional intakes calculated in INCA3. The SED (kcal/100 g) was defined as the ratio of total energy over the total weight consumed, including solid foods only. A low SED has been associated with good overall nutritional quality^(27)^. The MAR was calculated for each individual as the mean percentage of sex- and age-specific French RDA for twenty-two key nutrients. MAR values ranged from 0 % to 100 %, and the higher the MAR, the better the nutritional quality of the diet^(28)^. The PANDiet was calculated for each individual based on probabilities of adequacy for twenty-seven nutrients grouped into two sub-scores: the adequacy sub-score and the moderation sub-score. The adequacy sub-score assesses the probability of adequacy for food items whose usual intake should be above a reference value, whereas the moderation sub-score evaluates the probability of adequacy for food items whose usual intake should not exceed a reference value. PANDiet scores ranged from 0 to 100, where 100 represents 100 % of the usual intake adequacy for the twenty-seven nutrients^(21)^.

### Statistical analysis

ORCHID components, average daily ORCHID food group intake (in g/d), energy intake (kcal/d), total food intake (g/d), SED, MAR, PANDiet and nutrient intake were first presented across the ORCHID score quartiles, calculated for the whole sample and then stratified based on sex, given known dietary sex differences. The means of the score quartiles and their CI were calculated for each variable.

Because ORCHID food group intake and ORCHID components did not follow a normal distribution, non-parametric tests were used for these variables in analyses. Associations between the score and ORCHID components and the score and the ORCHID food group intake were assessed using Spearman correlations. Associations between the score and total food intake, energy intake, nutrient intake, SED, MAR and PANDiet were assessed using Pearson correlations. Wilcoxon tests were used to assess the effect of sex on ORCHID components and ORCHID food group intake, and the relative difference between the men and women means was calculated. Energy intake, nutrient intake, SED, MAR and PANDiet differences between sexes were assessed using analyses of variance adjusted on the score quartiles, and the absolute difference between men and women means was calculated.

In terms of correlations, we describe them as being of the following strengths: high strength (|*r*|>0·5), moderate strength (0·5>=|*r*|>0·2) or low strength (|*r*|<0·2).

Socio-demographic characteristics were described and statistically compared between the score quartiles using the *χ* ^2^test.

All means and SD are presented as follows: mean ± sd, and 95 % confidence intervals are presented as follows: (,). All analyses accounted for the complex INCA3 sampling frame design (R package (survey)^(29)^ except for the Spearman correlations and all analyses were performed using R, version R-4.1.2^(30)^.

## Results

### ORCHID score description and correlation with ORCHID components

The score was normally distributed. The average score was 53·5 (sd 17·8) and ranged from −36 to 115. The average score was not significantly different between women and men (53·7 (sd 16·5) and 52·6 (sd 19·1), respectively). The range of values was smaller for men (-13·5 to 99) as compared with women (-40 to 112).

Among the whole sample, three of the twenty components were positively correlated with the score with a high strength of correlation (online Supplementary Table 1), that is, the components ‘fruits’, ‘vegetables’ and ‘wholemeal or semi-wholemeal products’. Six components were positively correlated with the score with a moderate strength of correlation. The other components were either not correlated with the score or the correlation was of low strength.

No significant ORCHID component differences were found between men and women with the exception of ‘refined starches and potatoes’ (men had +2·25 points), ‘cheese’ (+0·61 point), ‘poultry’, ‘fatty fish’ and ‘oils’. The differences between men and women for the three last ORCHID components are less than 0·5 points (online Supplementary Table 2).

**Table 2.** Food group intakes by whole and by quartile of ORCHID score (*n* 696) (g/d) (Mean values and standard deviations; 95 % confidence intervals)

Food group intakes (g/d)	All				Q1		Q2		Q3		Q4	
	Mean	sd	Spearman correlation*	*p*-value Spearman*	Mean	95 % CI	Mean	95 % CI	Mean	95 % CI	Mean	95 % CI
Fruits	245·9	179·1	0·41	*P* < 0·001	169·3	132·0, 206·6	221·5	176·2, 266·7	268·9	234·3, 303·4	324·1	296·6, 351·7
Vegetables	162·3	120·2	0·38	*P* < 0·001	110·8	96·8, 124·7	156·9	128·9, 184·9	167·0	147·8, 186·2	214·4	187·3, 241·5
Wholemeal cereal products (including bread)	20·6	42·0	0·24	*P* < 0·001	19·4	11·2, 27·6	14·0	8·4, 19·7	18·5	11·6, 25·5	30·7	23·2, 38·1
Butter, margarine, fresh cream	17·3	16·3	-0·19	*P* < 0·001	22·6	18·4, 25·9	16·8	13·0, 20·6	15·2	12·6, 17·9	15·7	12·8, 17·5
Nuts	2·8	8·7	0·19	*P* < 0·001	3·2	0·5, 5·8	1·7	0·7, 2·7	1·9	1·0, 2·7	4·5	2·5, 6·5
Milk and fresh milk dairy	171·9	164·5	0·16	*P* < 0·001	127·4	101·8, 153·0	174·7	139·3, 210·1	167·8	127·7, 207·9	216·8	185·5, 248·0
Oils	7·2	9·0	0·15	*P* < 0·001	5·3	4·2, 6·4	8·3	6·3, 10·2	6·3	4·4, 8·3	8·7	6·8, 10·5
Lean fish and shellfish	22·7	35·6	0·10	0·007	18·4	12·2, 24·6	19·7	13·3, 26·1	28·1	20·3, 35·8	24·7	18·1, 31·2
Sweetened products (including sugar)	90·0	75·1	-0·10	0·006	104·6	85·1, 124·2	89·8	76·7, 102·9	78·1	64·4, 91·7	87·5	75·4, 99·6
Meat excluding poultry	83·3	57·5	-0·09	0·02	55·1	44·8, 65·5	60·7	49·5, 71·9	52·6	31·3, 74·0	49·9	42·4, 57·5
Refined starches (including bread) and potatoes	219·1	150·3	-0·08	0·04	225·8	196·6, 255·1	226·8	199·8, 253·8	236·9	168·2, 305·6	187·0	165·8, 208·2
Poultry (and rabbits)	28·6	34·3	0·07	0·06	26·8	19·6, 34·0	25·4	20·9, 30·0	28·2	19·0, 37·4	34·2	27·8, 40·6
Fatty fish	10·2	22·2	0·05	0·23	9·7	6·0, 13·4	9·9	5·6, 14·3	10·5	6·4, 14·5	10·8	7·3, 14·2
Eggs	16·2	23·4	-0·05	0·22	18·7	13·3, 24·0	16·7	11·3, 22·2	14·2	9·7, 18·6	15·0	11·4, 18·7
Deli meat excluding cooked ham	19·8	33·6	-0·05	0·16	26·1	18·2, 34·0	20·5	10·7, 30·3	16·6	9·9, 23·3	15·8	11·1, 20·5
Cooked ham	9·2	14·7	0·04	0·26	5·8	3·4, 8·3	11·4	7·4, 15·3	11·2	8·3, 14·1	8·3	5·5, 11·2
Sweetened drinks (including juice)	85·1	161·3	-0·04	0·25	120·8	77·2, 164·4	81·7	49·2, 114·2	69·5	35·1, 104·0	68·7	52·9, 84·5
Salted aperitif products	2·3	6·3	0·03	0·42	1·8	1·0, 2·7	2·9	1·2, 4·7	2·4	0·7, 4·2	1·9	1·0, 2·8
Legumes	7·1	22·0	-0·01	0·85	7·7	2·9, 12·5	7·3	3·6, 11·1	4·1	1·3, 6·9	9·0	4·5, 13·5
Cheese	38·6	32·1	0·00	0·97	35·8	29·3, 42·3	39·4	33·2, 45·5	41·2	35·0, 47·3	38·0	31·7, 44·3

* Tests of spearman correlation done without taking into account complex survey.

### Relationships between ORCHID food group intakes and the ORCHID score

Among the whole sample, the intakes of ‘fruits’, ‘vegetables’ and ‘wholemeal or semi-wholemeal products’ were positively correlated with the score with a moderate strength of correlation: for ‘fruits’, Q4 = 324·1 (296·6, 351·7) and Q1 = 169·3 (132·0, 206·6); for ‘vegetables’, Q4 = 214·4 (187·3, 241·5) and Q1 = 110·8 (96·8, 124·7) and for ‘wholemeal or semi-wholemeal Products’, Q4 = 30·7 (23·2, 38·1) and Q1 = 19·4 (11·2, 27·6) (Table 2). The intakes of ‘nuts’, ‘lean fish and shellfish’, ‘milk and fresh dairy products’ and ‘oils’ were positively correlated with the score with a low strength of correlation.

The intakes of ‘refined starches and potatoes’, ‘meat excluding poultry’, ‘sweetened products’ and ‘butter, margarine and fresh cream’ were negatively correlated with the score with a low strength of correlation. The score was not significantly associated with the consumption of other ORCHID food groups such as ‘legumes’.

The effect of sex was statistically significant for the intake of ‘refined starches and potatoes’ (men consume +50 g/d than women), ‘wholemeal or semi-wholemeal products’(men consume −1·8 g/d than women), ‘legumes’ (men consume −1·83 g/d than women), ‘cheese’ (+6·69 g/d), ‘butter, margarine and fresh cream’ (-1·53 g/d) and ‘lean fish and shellfish’ (the difference is negligible) (online Supplementary Table 3).

### Correlations between nutrient intakes, diet quality measures and the ORCHID score

Total food intake was significantly and positively associated with the ORCHID score, but energy intake was not. The ORCHID score was observed to be moderately and positively associated with fibres, proteins and good diet quality measures (PANDiet, adequacy sub-score and MAR). A negative association was found between the ORCHID score and SED, but no significant associations were found in terms of the moderation sub-score of the PANDiet or nutrients that should be limited, namely sugars, saturated fats and Na (Table 3).

**Table 3.** Diet quality indicators by whole and by quartile of ORCHID score (*n* 696) (Mean values and standard deviations; 95 % confidence intervals)

All					Q1		Q2		Q3		Q4	
Indicators	Mean	sd	Cor-Pearson	*p*-value Pearson	Mean	95 % CI	Mean	95 % CI	Mean	95 % CI	Mean	95 % CI
PANDiet	64·5	6·0	0·43	*P* < 0·001	61·2	60·1, 62·3	63·4	62·3, 64·4	66·0	64·8, 67·1	67·3	66·5, 68·1
SED (kcal/100 g)	141·4	33·8	-0·37	*P* < 0·001	158·0	149·3, 166·6	145·4	138·9, 151·8	134·0	127·6, 140·3	128·4	123,0 133·8
Fibres (g/d)	20·2	7·3	0·33	*P* < 0·001	17·4	15·9, 18·9	18·9	17·7, 20·0	21·1	18·8, 23·4	23·6	22·4, 24·8
Adequacy sub-score	66·3	16·1	0·31	*P* < 0·001	59·8	55·8, 63·9	63·9	60·6, 67·1	68·2	64·3, 72·1	73·2	71·0, 75·5
MAR (% adequacy)	84·6	10·7	0·30	*P* < 0·001	79·9	76·9, 82·9	83·4	81·3, 85·6	86·2	84·0, 88·3	89·1	87·8, 90·4
Total food intakes (g/d)	2698·4	809·5	0·18	*P* < 0·001	2534·5	2353·9, 2715·2	2606·8	2441·6, 2772·0	2754·4	2561·0, 2947·8	2900·1	2774·9, 3025·3
Proteins (g/kg/d)	1·1	0·4	0·12	*P* < 0·001	1·0	0·9, 1·1	1·1	1·0, 1·2	1·1	1·1, 1·2	1·2	1·1, 1·2
Na (mg/d)	3231·3	1418·0	0·07	0·06	3019·4	2711·3, 3327·5	3114·1	2820·7, 3407·6	3428·0	2997·3, 3858·6	3369·5	3142·3, 3596·6
Saturated fat (% energy)	29·9	13·7	-0·06	0·13	30·6	27·0, 34·1	29·4	26·5, 32·3	29·7	26·1, 33·3	30·0	27·8, 32·1
Sugars (g/d)	83·4	37·3	0·02	0·72	81·5	71·2, 91·9	81·4	74·8, 88·1	77·8	70·9, 84·7	92·6	87·1, 98·1
Energy intake (kcal/d)	1878·7	660·7	0·02	0·62	1846·8	1679·6, 2014·0	1837·2	1718·1, 1956·2	1893·3	1685·5, 2101·0	1938·8	1841·3, 2036·4
Moderation sub-score	62·7	11·9	0·02	0·67	62·7	59·7, 65·6	62·9	60·2, 65·5	63·8	61·0, 66·5	61·3	59·5, 63·2

SED, solid energy density; MAR, mean adequacy ratio.

Diet quality measures and nutrients are different depending on sex with the exception of the PANDiet and proteins when adjusted by the ORCHID score quartile. Men have lower moderation PANDiet sub-score than women (-10 %), higher scores in other diet quality measures than women and higher nutrient intakes than women (online Supplementary Table 4).

### Anthropometric and socio-demographic characteristics according to the ORCHID score quartile

The ORCHID score was significantly associated with educational level, smoking status, BMI and physical activity level. In the highest ORCHID score quartile, a larger proportion of individuals had a higher educational level and physical activity level compared with the other quartiles. The prevalence of current smokers was significantly higher in Q1 compared with Q4 of the ORCHID score. Sex, total monthly household income and the number of persons living in the household were not significantly associated with the ORCHID score (Table 4).

**Table 4.** Description of the 696 individuals of the sample by quartile of ORCHID score

	All	Q1	Q2	Q3	Q4	*P* value of *χ* ^2^ test
	%					
Number of individuals (60 years old and over)	696	168	162	160	206	
Sex						0·89
Man	42·10	49·07	36·42	38·13	44·13	
Woman	57·90	50·93	63·58	61·88	55·87	
Level of study						0·02
Primary + middle school	51·72	57·14	64·20	51·88	38·03	
High school	17·39	17·39	9·88	17·50	23·00	
Bachelor	17·82	14·91	14·81	16·25	23·47	
Master and over	13·07	10·56	11·11	14·38	15·49	
BMI (kg/m^2^)	27·25	27·69	27·16	27·66	26·53	
Body condition						0·02
Thinness	2·44	3·11	0·62	3·13	2·82	
Normal	36·93	31·68	37·04	33·75	43·19	
Overweight	40·23	39·13	40·12	45·00	37·56	
Obesity	20·11	26·09	22·22	16·88	16·43	
NA	0·29	0·00	0·00	1·25	0·00	
Total monthly household income per consumption unit (CU) in four classes						0·31
<900 €/month/CU	9·77	13·04	13·58	6·25	7·04	
900–1340 €/month/CU	18·10	21·12	22·22	14·38	15·49	
1340–1850 €/month/CU	21·70	21·74	17·90	25·63	21·60	
≥1850 €/month/CU	41·67	35·40	36·42	44·38	48·36	
NA	8·76	8·70	9·88	9·38	7·51	
Number of persons living in the household						0·18
1	33·91	37·89	32·72	38·13	28·64	
2	60·92	57·76	60·49	56·25	67·14	
3	4·60	3·73	4·94	5·63	4·23	
Smoking						0·02
Yes, daily	9·63	13·66	8·64	10·00	7·04	
Yes, sometimes	1·58	1·86	0·62	2·50	1·41	
No, but I have ever smoked	36·93	37·89	38·27	32·50	38·50	
No, never smoked	50·86	44·72	51·85	53·75	52·58	
NA	1·01	1·86	0·62	1·25	0·47	
Physical activity						0·05
Sedentary	31·18	40·37	30·86	31·25	24·41	
Active	58·62	52·17	58·64	60·63	61·97	
Very active	6·18	4·97	7·41	4·38	7·51	
NA	4·02	2·48	3·09	3·75	6·10	

## Discussion

This study describes an innovative and pragmatic healthy dietary diversity score that is easy to implement in intervention studies and adapted to the challenges of healthy ageing. Based on a sample of French individuals aged 60 years of age and over, we constructed an ORCHID score whose higher score was associated with higher consumption of the most healthful food groups and lower consumption of less healthful food groups. Furthermore, a higher ORCHID score was associated with a more nutritionally adequate diet, independent from energy intake. Construct validity was confirmed by expected relationships between the ORCHID score and socio-demographic characteristics: higher education, higher physical activity level, lower BMI and non-smoking status are significantly associated with higher ORCHID scores.

### ORCHID score validity

As no gold standard exists in nutrition, we chose to compare the ORCHID score to French scores, although neither is specific to older adults. Associations between the ORCHID score and ORCHID food group intakes went in the same direction as those reported in previous French studies validating nutritional measures, with higher ORCHID, PANDiet^(21)^ and PNNS-GS2^(5)^ (index based solely on French recommendations) scores all being correlated with higher fruit and vegetable intakes and lower processed meat intake. Our results were also consistent with a study validating the US Healthy Food Diversity Index, which is based on the concept of healthy diversity^(17)^.

As for Chaltiel *et al.* ^(5)^, certain ORCHID food groups (‘legumes’ and ‘nuts’) in our data were scarcely consumed, regardless of the diversity score. This explains why no associations between these ORCHID food groups and the ORCHID score were detected. In any case, incentives to increase ‘legume’ and ‘nut’ consumption would no doubt be beneficial to all older adults.

Associations between the ORCHID score and nutrient intakes were also generally the same as those reported in previous studies validating diet quality or diet diversity measures using data from French^(5)^, USA^(17)^, German^(18)^ or Danish^(31)^ surveys. Higher dietary diversity scores were associated with more favourable nutrient intakes. Regarding nutrients to limit, the German Healthy Food Diversity index was negatively associated with Na and sugars, but the associations were near zero^(18)^. The ORCHID score was not significantly associated with Na and sugars. The ORCHID score was not correlated with energy intake: a desirable attribute for a healthy diversity index given that higher diversity may solely reflect a higher energy intake^(32)^. We also found that higher ORCHID scores were correlated with diet quality measures including the SED (a proxy of low nutritional quality), the MAR (a measure of nutritional adequacy) and the PANDiet (a measure of overall nutritional quality). Taken together, these results underline the ORCHID score’s ability to reflect the nutritional quality of the diet.

Associations between the ORCHID score and socio-demographic and anthropometric characteristics, including educational level, physical activity level, BMI and smoking status, were similar to those reported for other scores in studies based on French dietary surveys^(5)^. On the other hand, certain socio-demographic characteristics that are typically linked to better dietary habits for older adults (i.e. being female as opposed to male, a higher total monthly household income and living with someone) were not associated with the ORCHID score^(25,33)^. Concerning sex, the PANDiet in the French population is not associated with sex^(21)^ and the ORCHID is well associated with the PANDiet (see Table 4). Moreover, men consume more energy, Na, sugars and fat but more fibres than women. Men consume more but in an unhealthy way compared with women, so they have a higher adequacy score but a lower moderation score (online Supplementary Table 4). This explains why they have the same PANDiet and ORCHID scores.

### A healthy dietary diversity score

Unlike most diversity scores, which are based on the number of food categories or foods consumed^(15)^, the ORCHID score is favourable to health given that (1) it relies on French nutrition recommendations for older adults^(19)^ and (2) it allows moderation dimension of the diet to be taken into account as shown by its negative correlation with the SED and its independence from energy. These results are in line with the healthy diversity scores developed by Drescher and Vadiveloo^(17,18)^.

### An easy-to-implement score

As opposed to other healthy dietary diversity scores requiring heavy data like portion weights^(14,15)^, the ORCHID score is based on consumption occurrences that can be easily reported, facilitating its implementation in public health intervention studies. For example, the Diversity ALAPAGE Score (an adaptation of the ORCHID score) was used in an intervention that aimed to increase the dietary diversity of older adults^(34)^.

### Healthy ageing

The ORCHID score is adapted to healthy ageing and the prevention of malnutrition: it focuses on protein intake by having eleven ORCHID components from ORCHID food groups about rich-protein food, with seven ORCHID components that are calculated only with the positive rating system, and promotes a limited consumption of energy-dense products often considered pleasant to consume^(20)^. The ORCHID score fills a gap by offering a healthy diversity score adapted to older adults and easy to collect in nutrition prevention interventions.

### Strengths and limitations

First, this study uses INCA3 data. Collected between 2014 and 2015, it is the most recent and comprehensive dietary data available in France. Food consumption patterns do not change rapidly over time^(35)^. Furthermore, the INCA3 data were of particular relevance for our study, as it provided us with a sample of adults aged 60 years and over at the national level. However, it should be noted that the recipes used to disaggregate complex dishes into ingredients were either standard or those most popular on the internet, meaning they may or may not correspond to the real consumption of study participants. The recipes were reduced only to their main ingredients. Nevertheless, the consumption of complex dishes was quite low among participants 60 years of age and over. Also, the FPQ is self-reported. Given that the population in question is of advanced age, participants may be more prone to memory issues. This could potentially affect data accuracy^(36)^. No correlation was found between the ORCHID score and total monthly household income and living with someone. However, it is offset by the others correlations found (smocking status, level of physical activity, level of study and BMI). Finally, the data did not include a biological sample.

Finally, there is no ‘gold’ standard against which the ORCHID score can be calibrated. However, the use of a detailed strategy based on other measures with converging results strengthened confidence in the ORCHID score’s reliability.

### Conclusion

The ORCHID score is the first healthy dietary diversity score developed to assess diet based on the rating of consumption occurrences. There is strong evidence that it will be a useful and pragmatic tool, that is, one that does not require overly sophisticated dietary data to assess dietary diversity and diet quality indicators. Not only is it the first healthy dietary diversity score for older adults, but it could also be easily adapted to other population groups through rating systems and food group modifications.

## Supporting information

Jacquemot et al. supplementary material 1 Jacquemot et al. supplementary material

Jacquemot et al. supplementary material 2 Jacquemot et al. supplementary material

Jacquemot et al. supplementary material 3 Jacquemot et al. supplementary material

Jacquemot et al. supplementary material 4 Jacquemot et al. supplementary material

Jacquemot et al. supplementary material 5 Jacquemot et al. supplementary material

Jacquemot et al. supplementary material 6 Jacquemot et al. supplementary material

Jacquemot et al. supplementary material 7 Jacquemot et al. supplementary material

Jacquemot et al. supplementary material 8 Jacquemot et al. supplementary material

## Supplementary material

For supplementary materials referred to in this article, please visit https://doi.org/10.1017/S0007114523002520
