# Supplementary material for: Development and validation of an occurrence-based healthy dietary diversity (ORCHID) score easy to operationalise in dietary prevention interventions in older adults: a French study
Source: Br J Nutr. 2023 Nov 8;131(6):1053–63. doi: 10.1017/S0007114523002520 (PMC10876453; doi:10.1017/S0007114523002520)
Supplement: Jacquemot et al. supplementary material 1 — Jacquemot et al. supplementary material [file S0007114523002520sup001.docx]

Supplemental material 1: Example of ORCHID Calculation of one individual

1) Calculation of the ORCHID components with positive rating system

| ALAPAGE food group | number of occurrences (nb_o) | Calculation method | ORCHID component (OC) |
| --- | --- | --- | --- |
| Fruits | 2 | - OC = nb_o*2 | 4 |
| Vegetables | 9 |  | 18 |
| Fatty Fish | 1 |  | 2 |
| Wholemeal and Semi-Wholemeal Products (Including Bread) | 1 |  | 2 |
| Legumes | 1 |  | 2 |
| Lean Fish and Shellfish | 2 |  | 4 |
| Nuts | 1 |  | 2 |
| Refined Starches (Including Bread) and Potatoes | 3 | - OC = nb_o | 3 |
| Poultry (and Rabbits) | 1 |  | 1 |
| Eggs | 1 |  | 1 |
| Cheese | 3 |  | 3 |
| Milk and Fresh Dairy Products | 0 |  | 0 |

2) Calcul of the ORCHID components with threshold rating system

| ALAPAGE food group | number of occurrences (nb_o) | Calculation method | ORCHID component (OC) |
| --- | --- | --- | --- |
| Meat excluding poultry | 2 | - nb_o =< 1,5, OC = nb_o*1 - nb_o> 1,5, OC = (nb_o- 1.5)*(-1) | -0.5 |
| Cooked Ham | 1 |  | 1 |
| Deli Meat Excluding Cooked Ham | 3 | - nb_o =<1,5, OC = nb_o - nb_o> 1,5, OC = (nb_o- 1.5)*(-2) | -1 |
| Butter, Margarine and Fresh cream | 4 | - nb_o =<3, OC = nb_o - nb_o> 3, OC = (nb_o- 3)*(-2) | -1 |
| Salted Aperitif Products | 0 |  | 0 |
| Sweetened Drinks (Including Juice) | 2 |  | 2 |
| Sweetened Products (Including Sugar) | 12 | - nb_o =<9, OC = nb_o - nb_o> 9, OC = (nb_o- 9)*(-2) | -6 |

3) Calculation of the ORCHID components Oils

| ALAPAGE food group | number of different type of oils | Calcul method | ORCHID component (OC) |
| --- | --- | --- | --- |
| Oils | - Olive oil - Mixed oil (colza and sunflower) | OC = ∑ different type of oil + ∑ (different mixed oil)*2 | 3 |

4) Calculation of ORCHID:

ORCHID = ∑ OC

**ORCHID = 39,5**
