# Supplementary material for: Development and validation of an occurrence-based healthy dietary diversity (ORCHID) score easy to operationalise in dietary prevention interventions in older adults: a French study
Source: Br J Nutr. 2023 Nov 8;131(6):1053–63. doi: 10.1017/S0007114523002520 (PMC10876453; doi:10.1017/S0007114523002520)
Supplement: Jacquemot et al. supplementary material 2 — Jacquemot et al. supplementary material [file S0007114523002520sup002.docx]

Supplemental Table 1 : ORCHID score by ORCHID component among the entire population and by quartile of ORCHID score (n=696)

|  | **ALL** | | | | **Q1** | | | **Q2** | | | **Q3** | | | **Q4** | | |
| --- | --- | --- | --- | --- | --- | --- | --- | --- | --- | --- | --- | --- | --- | --- | --- | --- |
| **ORCHID component** | **Mean** | **SD** | **Correlation of Spearman^a^** | **Spearman 𝝆-value** | **Mean** | **95% CI** | | **Mean** | **95% CI** | | **Mean** | **95% CI** | | **Mean** | **95% CI** | |
| Vegetables | 10.43 | 5.67 | 0.54 | < 0.001 | 7.01 | 6.23, | 7.79 | 9.26 | 8.09, | 10.43 | 11.42 | 10.43, | 12.40 | 14.05 | 12.93, | 15.17 |
| Fruits | 8.36 | 5.25 | 0.51 | < 0.001 | 5.77 | 4.83, | 6.72 | 7.03 | 6.05, | 8.00 | 9.08 | 8.19, | 9.97 | 11.58 | 10.72, | 12.44 |
| Wholemeal cereal products (including bread) | 7.60 | 7.70 | 0.50 | < 0.001 | 4.62 | 3.28, | 5.96 | 5.53 | 4.33, | 6.73 | 7.03 | 5.45, | 8.61 | 13.22 | 11.79, | 14.64 |
| Sweetened Products (Including Sugar) | 1.77 | 6.62 | 0.33 | < 0.001 | -1.10 | -3.61, | 0.21 | 1.67 | 0.61, | 2.74 | 3.69 | 2.84, | 4.54 | 3.41 | 2.43, | 4.39 |
| Nuts | 2.34 | 2.76 | 0.30 | < 0.001 | 1.80 | 1.47, | 2.13 | 1.96 | 1.66, | 2.25 | 1.79 | 1.51, | 2.07 | 3.82 | 3.16, | 4.48 |
| Lean Fish and Shellfish | 3.53 | 2.92 | 0.25 | < 0.001 | 3.03 | 2.54, | 3.52 | 2.89 | 2.53, | 3.25 | 3.66 | 3.06, | 4.26 | 4.55 | 3.82, | 5.28 |
| Poultry (and Rabbits) | 1.75 | 1.04 | 0.22 | < 0.001 | -0.07 | 1.26, | 1.73 | -0.17 | 1.38, | 1.88 | -0.04 | 1.58, | 2.22 | 0.04 | 1.77, | 2.18 |
| Butter, Margarine and Fresh cream | -1.21 | 4.33 | 0.22 | < 0.001 | -3.00 | -4.17, | -1.82 | -0.90 | -1.52, | -0.28 | -0.96 | -1.68, | -0.23 | 0.00 | -0.65, | 0.64 |
| Milk and Fresh Dairy Products | 3.02 | 2.54 | 0.20 | < 0.001 | 2.16 | 1.81, | 2.50 | 2.94 | 2.45, | 3.44 | 3.15 | 2.56, | 3.73 | 3.84 | 3.31, | 4.37 |
| Oils | 0.79 | 0.88 | 0.17 | < 0.001 | 0.67 | 0.49, | 0.86 | 0.86 | 0.69, | 1.03 | 0.62 | 0.44, | 0.80 | 0.98 | 0.83, | 1.13 |
| Eggs | 1.70 | 1.25 | 0.13 | < 0.001 | 1.38 | 1.19, | 1.57 | 1.76 | 1.48, | 2.05 | 1.68 | 1.46, | 1.90 | 1.96 | 1.69, | 2.22 |
| Fatty Fish | 0.77 | 1.26 | 0.12 | 0.001 | 0.69 | 0.40, | 0.99 | 0.53 | 0.31, | 0.76 | 0.84 | 0.56, | 1.13 | 1.01 | 0.67, | 1.35 |
| Sweetened Drinks (Including Juice) | -0.19 | 3.75 | 0.12 | 0.001 | -1.40 | -2.61, | -0.18 | -0.30 | -0.99, | 0.39 | 0.40 | 0.08, | 0.72 | 0.55 | 0.34, | 0.75 |
| Legumes | 2.16 | 1.55 | 0.12 | 0.002 | 1.82 | 1.65, | 1.99 | 2.14 | 1.66, | 2.62 | 2.10 | 1.80, | 2.39 | 2.57 | 2.23, | 2.92 |
| Other deli meat | -0.17 | 1.33 | 0.06 | 0.09 | -0.43 | -0.77, | -0.08 | -0.10 | -0.35, | 0.15 | -0.10 | -0.50, | 0.29 | -0.07 | -0.33, | 0.19 |
| Meat excluding poultry | -0.06 | 1.31 | 0.06 | 0.09 | 1.49 | -0.29, | 0.14 | 1.63 | -0.43, | 0.09 | 1.90 | -0.41, | 0.34 | 1.90 | -0.13, | 0.21 |
| Cheese | 2.71 | 2.01 | 0.05 | 0.18 | 2.45 | 1.99, | 2.92 | 2.65 | 2.28, | 3.03 | 2.93 | 2.56, | 3.30 | 2.79 | 2.37, | 3.22 |
| Cooked Ham | 0.17 | 0.60 | 0.03 | 0.49 | 0.13 | 0.04, | 0.22 | 0.10 | -0.09, | 0.28 | 0.28 | 0.12, | 0.45 | 0.17 | 0.09, | 0.26 |
| Refined Starches (Including Bread) and Potatoes | 7.83 | 3.91 | 0.02 | 0.58 | 7.54 | 6.81, | 8.27 | 7.93 | 7.30, | 8.55 | 8.39 | 6.90, | 9.87 | 7.49 | 6.75, | 8.24 |
| Salted Aperitif Products | 0.18 | 0.52 | 0.01 | 0.85 | 0.18 | 0.07, | 0.28 | 0.18 | 0.05, | 0.31 | 0.16 | 0.07, | 0.26 | 0.21 | 0.10, | 0.31 |
| a tests of spearman correlation done without taking into account complex survey, CI = confidence interval | | | | | | | | | | | | | | | | |
