# Supplementary material for: Development and validation of an occurrence-based healthy dietary diversity (ORCHID) score easy to operationalise in dietary prevention interventions in older adults: a French study
Source: Br J Nutr. 2023 Nov 8;131(6):1053–63. doi: 10.1017/S0007114523002520 (PMC10876453; doi:10.1017/S0007114523002520)
Supplement: Jacquemot et al. supplementary material 3 — Jacquemot et al. supplementary material [file S0007114523002520sup003.docx]

Supplemental Table 2: ORCHID by ORCHID component and by sex (n= 696)

| **ORCHID component** |  |  | **Men** | | | | | | | **Quartiles of ORCHID among Men (n= 293)** | | | | | | | | | | | | |
| --- | --- | --- | --- | --- | --- | --- | --- | --- | --- | --- | --- | --- | --- | --- | --- | --- | --- | --- | --- | --- | --- | --- |
|  |  |  |  | |  | |  | |  | **Q1** | | | | **Q2** | | | **Q3** | | | **Q4** | | |
|  |  | **ORCHID range** | [-13.5;103] | | | | | | | [-13.5;41.5[ | | | | [41.5;52[ | | | [52;64[ | | | [64;103[ | | |
|  |  | **n=** | 293 | | | | | | | 77 | | |  | 58 | | | 68 | | | 90 | | |
|  | **Wilcoxon test Score vs sexe p-value sex** | **relative difference in means** | **Mean** | **SD** | | **Correlation of Spearman^a^** | | **p-value Spearman^a^** | | **Mean** | **95% CI** | | | **Mean** | **95% CI** | | **Mean** | **95% CI** | | **Mean** | **95% CI** | |
| Refined Starches (Including Bread) and Potatoes | < 0.001 | 2.25 | 8.97 | 4.00 | | 0.08 | | 0.18 | | 8.74 | 7.78, | 9.69 | | 8.29 | 7.33, | 9.25 | 10.16 | 7.78, | 12.55 | 8.66 | 7.61, | 9.71 |
| Cheese | < 0.001 | 0.61 | 3.02 | 1.96 | | 0.05 | | 0.43 | | 2.55 | 2.08, | 3.01 | | 2.80 | 2.13, | 3.47 | 3.48 | 3.11, | 3.86 | 3.23 | 2.63, | 3.83 |
| Poultry (and Rabbits) | 0.004 | -0.11 | 1.69 | 1.18 | | 0.02 | | 0.78 | | 1.57 | 1.23, | 1.91 | | 1.55 | 1.18, | 1.93 | 1.85 | 1.31, | 2.39 | 1.80 | 1.49, | 2.11 |
| Oils | 0.04 | -0.19 | 0.69 | 0.85 | | 0.16 | | 0.01 | | 0.57 | 0.39 | 0.76 | | 0.71 | 0.38, | 1.03 | 0.64 | 0.32, | 0.96 | 0.85 | 0.63, | 1.06 |
| Fatty Fish | 0.05 | 0.24 | 0.89 | 1.43 | | 0.10 | | 0.07 | | 0.88 | 0.40, | 1.36 | | 0.61 | 0.22, | 1.01 | 0.89 | 0.43, | 1.35 | 1.16 | 0.66, | 1.66 |
| Eggs | 0.08 | -0.28 | 1.55 | 1.05 | | 0.07 | | 0.26 | | 1.42 | 1.18, | 1.66 | | 1.49 | 1.22, | 1.77 | 1.60 | 1.19, | 2.02 | 1.71 | 1.44, | 1.97 |
| Lean Fish and Shellfish | 0.16 | -0.72 | 3.16 | 2.35 | | 0.23 | | < 0.001 | | 2.85 | 2.32, | 3.38 | | 2.42 | 1.83, | 3.02 | 2.98 | 2.50, | 3.46 | 4.39 | 3.54, | 5.24 |
| Milk and Fresh Dairy Products | 0.18 | -0.29 | 2.88 | 2.59 | | 0.19 | | 0.001 | | 2.17 | 1.56, | 2.78 | | 2.69 | 1.94, | 3.45 | 2.62 | 1.76, | 3.48 | 4.04 | 3.24, | 4.83 |
| Legumes | 0.21 | 0.06 | 2.19 | 1.35 | | 0.15 | | 0.008 | | 1.78 | 1.50, | 2.06 | | 2.10 | 1.92, | 2.27 | 2.32 | 1.88, | 2.76 | 2.57 | 2.12, | 3.01 |
| Meat excluding poultry | 0.40 | -0.39 | -0.26 | 1.26 | | 0.11 | | 0.05 | | -0.22 | -0.57, | 0.13 | | -0.50 | -0.99, | -0.02 | -0.17 | -0.86, | 0.53 | -0.16 | -0.48, | 0.15 |
| Cooked Ham | 0.43 | -0.08 | 0.13 | 0.60 | | 0.02 | | 0.80 | | 0.10 | 0.01, | 0.19 | | -0.11 | -0.49, | 0.28 | 0.39 | 0.14, | 0.64 | 0.14 | 0.04, | 0.24 |
| Sweetened Drinks (Including Juice) | 0.43 | -0.59 | -0.49 | 4.01 | | 0.13 | | 0.02 | | -1.93 | -4.10, | 0.25 | | -0.38 | -1.34, | 0.58 | -0.13 | -0.94, | 0.69 | 0.51 | 0.20, | 0.83 |
| Other deli meat | 0.51 | -0.34 | -0.34 | 1.64 | | 0.06 | | 0.34 | | -0.66 | -1.19, | -0.13 | | -0.26 | -0.77, | 0.24 | -0.30 | -1.04, | 0.45 | -0.14 | -0.65 | 0.37 |
| Nuts | 0.57 | -0.36 | 2.16 | 2.09 | | 0.25 | | < 0.001 | | 1.77 | 1.29, | 2.25 | | 1.98 | 1.64, | 2.31 | 1.88 | 1.65, | 2.12 | 3.02 | 2.32, | 3.73 |
| Sweetened Products (Including Sugar) | 0.61 | -0.96 | 1.28 | 7.31 | | 0.36 | | < 0.001 | | -2.15 | -5.14, | 0.85 | | 0.75 | -1.31, | 2.82 | 3.90 | 2.54, | 5.26 | 2.66 | 0.88, | 4.43 |
| Butter, Margarine and Fresh cream | 0.61 | 0.04 | -1.19 | 3.97 | | 0.28 | | < 0.001 | | -3.12 | -4.69, | -1.54 | | -0.80 | -1.53, | -0.08 | -1.08 | -1.88 | -0.27 | 0.33 | -0.55, | 1.21 |
| Vegetables | 0.62 | 0.59 | 10.73 | 6.29 | | 0.56 | | < 0.001 | | 6.70 | 5.40, | 8.00 | | 9.31 | 7.17, | 11.45 | 12.17 | 10.66, | 13.68 | 14.79 | 12.85, | 16.72 |
| Salted Aperitif Products | 0.7238 | 0.02 | 0.19 | 0.57 | | 0.11 | | 0.06 | | 0.22 | 0.04 | 0.41 | | 0.05 | -0.14, | 0.25 | 0.20 | 0.04, | 0.37 | 0.29 | 0.10, | 0.48 |
| Fruits | 0.86 | 0.09 | 8.41 | 5.55 | | 0.51 | | < 0.001 | | 5.40 | 3.92, | 6.88 | | 7.20 | 5.64, | 8.76 | 8.95 | 7.74, | 10.17 | 12.10 | 10.62, | 13.58 |
| Wholemeal cereal products (including bread) | 0.92 | -0.05 | 7.57 | 8.03 | | 0.46 | | < 0.001 | | 4.63 | 2.44, | 6.82 | | 6.97 | 4.70, | 9.23 | 5.64 | 3.56, | 7.72 | 13.15 | 10.88, | 15.41 |

Supplemental Table 2 continued: ORCHID by ORCHID component and by sex (n= 696)

|  | **Women** | | | | **Q1** | | | **Q2** | | | **Q3** | | | **Q4** |  |  |
| --- | --- | --- | --- | --- | --- | --- | --- | --- | --- | --- | --- | --- | --- | --- | --- | --- |
| ORCHID range | [-36;115] | | | | [-36;43.5[ | | | [43.5;53.5[ | | | [53.5;63.4[ | | | [63.4;115] |  |  |
| n= | 403 | | | | 94 | | | 99 | | | 96 | | | 114 |  |  |
| **ORCHID component** | **Mean** | **SD** | **Correlation of Spearman^a^** | **𝝆-value Spearman^a^** | **Mean** | **95% CI** | | **Mean** | **95% CI** | | **Mean** | **95% CI** | | **Mean** | **95% CI** | |
| Vegetables | 10.14 | 5.00 | 0.53 | < 0.001 | 7.21 | 6.33, | 8.09 | 9.25 | 7.95, | 10.54 | 10.83 | 9.65, | 12.02 | 13.29 | 12.20, | 14.38 |
| Wholemeal cereal products (including bread) | 7.62 | 7.37 | 0.53 | < 0.001 | 4.36 | 2.96, | 5.77 | 4.67 | 3.22, | 6.11 | 8.09 | 5.81, | 10.38 | 13.40 | 11.62, | 15.19 |
| Fruits | 8.31 | 4.94 | 0.50 | < 0.001 | 6.18 | 5.06, | 7.31 | 6.81 | 5.57, | 8.05 | 9.08 | 8.14, | 10.01 | 11.20 | 10.13, | 12.27 |
| Nuts | 2.52 | 3.27 | 0.33 | < 0.001 | 1.74 | 1.41, | 2.06 | 2.09 | 1.54, | 2.63 | 1.69 | 1.22, | 2.16 | 4.62 | 3.52, | 5.71 |
| Meat excluding poultry | 0.13 | 1.36 | 0.30 | < 0.001 | 0.12 | -0.10, | 0.33 | 0.01 | -0.20, | 0.22 | 0.14 | -0.08, | 0.37 | 0.25 | 0.09, | 0.41 |
| Sweetened Products (Including Sugar) | 2.24 | 5.84 | 0.30 | < 0.001 | -0.70 | -2.85, | 1.45 | 1.91 | 0.79, | 3.03 | 3.52 | 2.63, | 4.40 | 4.22 | 3.42, | 5.03 |
| Lean Fish and Shellfish | 3.88 | 3.36 | 0.26 | < 0.001 | 3.09 | 2.24, | 3.93 | 3.45 | 2.90, | 3.99 | 4.25 | 3.27, | 5.22 | 4.76 | 3.48, | 6.03 |
| Milk and Fresh Dairy Products | 3.17 | 2.48 | 0.21 | < 0.001 | 2.03 | 1.54, | 2.51 | 3.32 | 2.68, | 3.96 | 3.74 | 3.23, | 4.26 | 3.57 | 2.88, | 4.27 |
| Eggs | 1.84 | 1.40 | 0.18 | < 0.001 | 1.41 | 1.10, | 1.73 | 1.99 | 1.49, | 2.49 | 1.76 | 1.44, | 2.09 | 2.19 | 1.74, | 2.64 |
| Oils | 0.88 | 0.89 | 0.17 | < 0.001 | 0.87 | 0.57, | 1.17 | 0.92 | 0.72, | 1.13 | 0.63 | 0.46, | 0.80 | 1.09 | 0.88, | 1.31 |
| Butter, Margarine and Fresh cream | -1.23 | 4.66 | 0.17 | < 0.001 | -2.49 | -4.33, | -0.65 | -1.31 | -2.36, | -0.27 | -0.76 | -1.82, | 0.29 | -0.34 | -1.22, | 0.54 |
| Fatty Fish | 0.65 | 1.06 | 0.15 | 0.004 | 0.46 | 0.16, | 0.76 | 0.55 | 0.31, | 0.80 | 0.73 | 0.42, | 1.03 | 0.85 | 0.46, | 1.24 |
| Sweetened Drinks (Including Juice) | 0.10 | 3.47 | 0.11 | 0.02 | -0.78 | -1.91, | 0.36 | 0.01 | -0.60, | 0.62 | 0.58 | 0.16, | 0.99 | 0.61 | 0.30, | 0.92 |
| Poultry (and Rabbits) | 1.80 | 0.84 | 0.11 | 0.03 | 1.42 | 1.11, | 1.73 | 1.73 | 1.33, | 2.14 | 1.89 | 1.53, | 2.24 | 2.17 | 1.84, | 2.50 |
| Legumes | 2.13 | 1.72 | 0.09 | 0.08 | 1.83 | 1.64, | 2.02 | 2.23 | 1.25, | 3.20 | 1.93 | 1.59, | 2.27 | 2.54 | 2.02, | 3.06 |
| Other deli meat | -0.01 | 0.93 | 0.07 | 0.16 | -0.15 | -0.57, | 0.26 | 0.03 | -0.18, | 0.24 | 0.09 | -0.07, | 0.26 | 0.00 | -0.16, | 0.16 |
| Cheese | 2.40 | 2.01 | 0.07 | 0.17 | 2.34 | 1.59, | 3.10 | 2.44 | 1.94, | 2.94 | 2.49 | 1.97, | 3.01 | 2.34 | 1.86, | 2.82 |
| Cooked Ham | 0.21 | 0.60 | 0.03 | 0.53 | 0.16 | -0.01, | 0.34 | 0.29 | 0.13, | 0.44 | 0.17 | 0.00, | 0.34 | 0.21 | 0.06, | 0.37 |
| Refined Starches (Including Bread) and Potatoes | 6.73 | 3.49 | -0.01 | 0.80 | 6.28 | 5.32, | 7.24 | 7.55 | 6.78, | 8.32 | 6.78 | 5.87, | 7.70 | 6.30 | 5.39, | 7.21 |
| Salted Aperitif Products | 0.17 | 0.48 | -0.09 | 0.09 | 0.13 | 0.04, | 0.21 | 0.32 | 0.15, | 0.50 | 0.12 | 0.00, | 0.23 | 0.12 | 0.00, | 0.23 |
| ^a^  tests of spearman correlation done without taking into account complex survey CI = confidence interval | | | | | | | | |  |  |  |  |  |  |  |  |
