# Supplementary material for: Development and validation of an occurrence-based healthy dietary diversity (ORCHID) score easy to operationalise in dietary prevention interventions in older adults: a French study
Source: Br J Nutr. 2023 Nov 8;131(6):1053–63. doi: 10.1017/S0007114523002520 (PMC10876453; doi:10.1017/S0007114523002520)
Supplement: Jacquemot et al. supplementary material 4 — Jacquemot et al. supplementary material [file S0007114523002520sup004.docx]

Supplemental Table 3: Food group intakes by ORCHID score quartiles and by sex (n= 696)

|  | **Men** | | | | | | **ORCHID quartiles among men (n= 293 )** | | | | | | | | | | | |
| --- | --- | --- | --- | --- | --- | --- | --- | --- | --- | --- | --- | --- | --- | --- | --- | --- | --- | --- |
|  |  |  |  |  |  |  | **Q1** | | | **Q2** | | | **Q3** | | | **Q4** | | |
|  | **ORCHID score range** | | [-13,5;103] | | | | [-13.5;41.5[ | | | [41.5;52[ | | | [52;64[ | | | [64;103[ | | |
|  | **n=** | | 293 | | | | 77 | | | 58 | | | 68 | | | 90 | | |
| **Food group intakes (g/day)** | **wilcoxon  p-value sex** | **relative difference in means (g/day)** | **Mean** | **SD** | **Spearman^a^ Correlation** | **Spearman^a^ p-value** | **Mean** | **95% CI** | | **Mean** | **95% CI** | | **Mean** | **95% CI** | | **Mean** | **95% CI** | |
| Refined Starches (Including Bread) and Potatoes | < 0.001 | 50.61 | 269.7 | 171.6 | -0.08 | 0.19 | 275.7 | 234.5, | 316.9 | 270.1 | 214.5, | 325.7 | 301.3 | 185.1, | 417.5 | 230.9 | 195.3, | 266.5 |
| Lean Fish and Shellfish | < 0.001 | 0.23 | 22.9 | 37.0 | 0.07 | 0.22 | 22.8 | 13.2, | 32.5 | 20.6 | 10.3, | 30.8 | 24.1 | 11.3, | 36.9 | 23.9 | 14.4, | 33.4 |
| Cheese | < 0.001 | 6.69 | 45.3 | 32.9 | -0.05 | 0.36 | 40.9 | 32.8, | 49.1 | 43.2 | 32.0, | 54.3 | 51.1 | 44.6, | 57.7 | 45.9 | 35.9, | 55.9 |
| Legumes | 0.01 | 1.83 | 8.9 | 26.8 | -0.01 | 0.85 | 10.4 | 1.0, | 19.7 | 5.6 | 1.8, | 9.3 | 5.7 | 0.6, | 10.9 | 13.7 | 5.7, | 21.7 |
| Wholemeal cereal Products (Including Bread) | 0.01 | -1.83 | 18.8 | 42.7 | 0.22 | < 0.001 | 13.6 | 5.1, | 22.1 | 17.7 | 5.3, | 30.1 | 15.2 | 5.3, | 25.1 | 28.9 | 18.5, | 39.3 |
| Butter, Margarine and Fresh cream | 0.03 | 1.53 | 18.9 | 15.7 | -0.27 | < 0.001 | 25.1 | 20.1, | 30.1 | 16.3 | 11.6, | 21.0 | 17.7 | 14.0, | 21.4 | 16.0 | 12.2, | 19.8 |
| Cooked Ham | 0.19 | -0.05 | 9.1 | 15.4 | 0.09 | 0.12 | 5.0 | 1.3, | 8.7 | 12.9 | 5.5, | 20.2 | 11.2 | 6.6, | 15.8 | 7.8 | 3.2, | 12.3 |
| Oils | 0.20 | 0.04 | 7.19 | 9.64 | 0.13 | 0.03 | 5.4 | 3.6, | 7.2 | 8.2 | 4.7, | 11.8 | 6.6 | 3.3, | 9.9 | 8.6 | 5.6, | 11.6 |
| Eggs | 0.20 | 0.06 | 16.2 | 25.9 | -0.11 | 0.07 | 21.6 | 12.9, | 30.4 | 14.3 | 4.3, | 24.3 | 15.0 | 8.0, | 22.0 | 13.8 | 8.2, | 19.4 |
| Vegetables | 0.24 | 12.95 | 175.3 | 135.5 | 0.39 | < 0.001 | 114.9 | 89.7, | 140.0 | 164.8 | 108.3, | 221.2 | 185.9 | 152.3, | 219.4 | 236.9 | 194.4, | 279.3 |
| Fruits | 0.24 | 13.59 | 259.4 | 195.5 | 0.36 | < 0.001 | 181.9 | 116.6, | 247.2 | 233.1 | 147.8, | 318.4 | 293.7 | 241.4, | 345.9 | 329.8 | 293.5, | 366.1 |
| Sweetened Products (Including Sugar) | 0.25 | 5.27 | 95.3 | 85.4 | -0.18 | 0.002 | 114.6 | 79.7, | 149.5 | 90.9 | 63.6, | 118.3 | 91.7 | 67.8, | 115.5 | 83.3 | 66.6, | 100.0 |
| Poultry (and Rabbits) | 0.45 | 1.46 | 30.1 | 35.6 | 0.11 | 0.06 | 28.2 | 16.4, | 40.0 | 23.1 | 16.4, | 29.9 | 31.1 | 15.6, | 46.7 | 37.7 | 27.5, | 47.9 |
| Meat excluding poultry | 0.48 | 12.73 | 67.4 | 59.5 | -0.08 | 0.15 | 69.5 | 53.3, | 85.7 | 73.7 | 53.6, | 93.8 | 61.3 | 21.3, | 101.4 | 65.3 | 53.5, | 77.1 |
| Salted Aperitif Products | 0.51 | 0.28 | 2.6 | 7.2 | 0.07 | 0.23 | 2.1 | 0.7, | 3.5 | 2.5 | -0.8, | 5.7 | 3.2 | -0.3, | 6.7 | 2.5 | 0.9, | 4.1 |
| Sweetened Drinks (Including Juice) | 0.61 | 13.05 | 98.2 | 175.8 | -0.09 | 0.11 | 150.7 | 74.6, | 226.9 | 97.1 | 51.3, | 142.8 | 67.6 | 25.6, | 109.6 | 76.2 | 47.8, | 104.6 |
| Fatty Fish | 0.77 | -0.28 | 9.9 | 21.9 | 0.05 | 0.38 | 6.6 | 2.6, | 10.6 | 8.3 | 1.5, | 15.1 | 13.8 | 6.1, | 21.5 | 11.1 | 5.2, | 16.9 |
| Milk and Fresh Dairy Products | 0.92 | -5.25 | 166.6 | 171.4 | 0.14 | 0.02 | 129.2 | 88.8, | 169.7 | 168.6 | 115.5, | 221.6 | 139.1 | 75.3, | 202.9 | 231.1 | 184.7, | 277.6 |
| Nuts | 0.97 | 0.47 | 3.3 | 10.1 | 0.09 | 0.13 | 5.0 | -0.2, | 10.1 | 1.4 | 0.2, | 2.6 | 2.1 | 0.6, | 3.6 | 4.6 | 1.8, | 7.3 |
| Other deli meat | 0.98 | 3.94 | 23.7 | 30.3 | -0.10 | 0.10 | 33.4 | 21.6, | 45.2 | 18.6 | 9.9, | 27.2 | 20.2 | 9.3, | 31.1 | 22.2 | 13.9, | 30.5 |

Supplemental Table 3 continued: Food group intakes by ORCHID score quartiles and by sex (n= 696)

| **Women** | | | | | **ORCHID quartiles among women(n= 403 )** | | | | | | | | | | | |
| --- | --- | --- | --- | --- | --- | --- | --- | --- | --- | --- | --- | --- | --- | --- | --- | --- |
|  |  |  |  |  | **Q1** | | | **Q2** | | | **Q3** | | | **Q4** | | |
| **ORCHID Range** | [-36;115] | | | | [-36;43,5[ | | | [43,5;53,5[ | | | [53,5;63,4[ | | | [63,4;115] | | |
| **N=** | 403 | | | | 94 | | | 99 | | | 96 | | | 114 | | |
| **Food group intakes (g/day)** | **Mean** | **SD** | **Correlation of Spearman^a^** | **p-value Spearman^a^** | **Mean** | **95% CI** | | **Mean** | **95% CI** | | **Mean** | **95% CI** | | **Mean** | **95% CI** | |
| Fruits | 245.9 | 179.1 | 0.45 | < 0.001 | 168.1 | 126.3 | 182.3 | 223.0 | 167.1 | 259.8 | 268.2 | 209.5 | 277.9 | 325.0 | 278.0 | 362.4 |
| Vegetables | 162.3 | 120.2 | 0.38 | < 0.001 | 108.7 | 86.6 | 118.4 | 158.9 | 126.7 | 180.0 | 169.4 | 134.2 | 173.0 | 213.4 | 159.3 | 221.3 |
| Nuts | 2.8 | 8.7 | 0.27 | < 0.001 | 3.1 | 0.2 | 2.2 | 1.8 | 0.5 | 3.8 | 1.9 | 0.7 | 2.8 | 4.4 | 1.7 | 6.9 |
| Wholemeal cereal Products (Including Bread) | 20.6 | 42.0 | 0.25 | < 0.001 | 19.2 | 10.7 | 39.1 | 14.5 | 7.2 | 16.0 | 17.8 | 9.8 | 30.7 | 30.9 | 21.5 | 44.4 |
| Oils | 7.2 | 9.0 | 0.18 | < 0.001 | 5.6 | 4.2 | 7.2 | 8.2 | 6.2 | 10.2 | 6.2 | 4.3 | 7.4 | 8.7 | 6.5 | 11.1 |
| Milk and Fresh Dairy Products | 171.9 | 164.5 | 0.18 | < 0.001 | 123.8 | 85.1 | 151.6 | 176.5 | 143.2 | 224.9 | 174.2 | 171.3 | 244.9 | 214.1 | 154.0 | 240.7 |
| Lean Fish and Shellfish | 22.7 | 35.6 | 0.13 | 0.009 | 18.2 | 4.3 | 22.8 | 20.5 | 11.9 | 29.0 | 27.0 | 19.1 | 40.4 | 24.9 | 16.7 | 34.9 |
| Butter, Margarine and Fresh cream | 17.3 | 16.3 | -0.12 | 0.02 | 21.8 | 12.8 | 24.4 | 17.4 | 11.9 | 24.8 | 14.8 | 8.9 | 15.2 | 15.2 | 11.3 | 17.6 |
| Meat excluding poultry | 54.6 | 51.7 | -0.09 | 0.06 | 53.1 | 26.7 | 47.0 | 62.9 | 40.6 | 64.7 | 53.5 | 37.3 | 54.3 | 49.4 | 26.0 | 41.3 |
| Refined Starches (Including Bread) and Potatoes | 219.1 | 150.3 | -0.08 | 0.12 | 224.2 | 133.1 | 212.7 | 232.5 | 173.8 | 220.1 | 232.8 | 138.5 | 194.8 | 186.5 | 125.8 | 159.6 |
| Cheese | 38.6 | 32.1 | 0.06 | 0.25 | 36.0 | 20.8 | 41.3 | 38.7 | 27.9 | 41.2 | 41.7 | 24.1 | 41.0 | 38.0 | 23.0 | 37.3 |
| Poultry (and Rabbits) | 28.6 | 34.3 | 0.04 | 0.40 | 28.9 | 18.7 | 40.4 | 22.4 | 16.5 | 27.0 | 28.6 | 16.6 | 35.8 | 34.5 | 22.5 | 40.3 |
| Fatty Fish | 10.2 | 22.2 | 0.04 | 0.48 | 9.0 | 6.0 | 16.9 | 11.2 | 7.0 | 20.8 | 10.1 | 3.2 | 9.7 | 10.7 | 6.2 | 14.3 |
| Sweetened Products (Including Sugar) | 90.0 | 75.1 | -0.04 | 0.47 | 101.9 | 72.3 | 106.3 | 93.5 | 81.5 | 110.4 | 78.1 | 52.0 | 78.0 | 86.6 | 73.3 | 106.7 |
| Other deli meat | 19.8 | 33.6 | -0.02 | 0.70 | 25.5 | 8.8 | 26.6 | 21.4 | 5.7 | 42.2 | 16.2 | 5.5 | 19.1 | 16.0 | 6.0 | 13.5 |
| Salted Aperitif Products | 2.3 | 6.3 | -0.01 | 0.92 | 1.8 | 0.6 | 2.3 | 3.1 | 1.6 | 5.7 | 2.4 | 0.5 | 2.6 | 1.9 | 0.5 | 2.2 |
| Legumes | 7.1 | 22.0 | 0.00 | 0.94 | 7.2 | 0.9 | 7.4 | 7.4 | 2.6 | 15.7 | 4.5 | 0.8 | 6.0 | 9.1 | -0.6 | 9.6 |
| Eggs | 16.2 | 23.4 | 0.00 | 0.97 | 18.1 | 9.8 | 19.1 | 17.6 | 14.4 | 26.9 | 14.0 | 7.4 | 18.6 | 15.1 | 12.0 | 20.8 |
| Cooked Ham | 9.2 | 14.7 | 0.00 | 0.98 | 5.7 | 3.5 | 9.2 | 12.0 | 6.9 | 15.4 | 10.8 | 7.2 | 13.7 | 8.4 | 4.9 | 13.3 |
| Sweetened Drinks (Including Juice) | 85.1 | 161.3 | 0.00 | 0.93 | 114.7 | 37.2 | 120.3 | 80.2 | 39.4 | 89.0 | 77.4 | 32.9 | 140.7 | 67.7 | 42.4 | 76.4 |
| ^a^  tests of spearman correlation done without taking into account complex survey CI = confidence interval | | | | | | | | | | |  |  |  |  |  |  |
