# Supplementary material for: Development and validation of an occurrence-based healthy dietary diversity (ORCHID) score easy to operationalise in dietary prevention interventions in older adults: a French study
Source: Br J Nutr. 2023 Nov 8;131(6):1053–63. doi: 10.1017/S0007114523002520 (PMC10876453; doi:10.1017/S0007114523002520)
Supplement: Jacquemot et al. supplementary material 5 — Jacquemot et al. supplementary material [file S0007114523002520sup005.docx]

Supplemental table 4: Relations between nutrient intake, diet quality measures and ORCHID by sex

|  |  |  | **Men** | | | | **ORCHID quartiles among Men (n=293)** | | | | | | | | | | | |
| --- | --- | --- | --- | --- | --- | --- | --- | --- | --- | --- | --- | --- | --- | --- | --- | --- | --- | --- |
|  |  |  |  |  |  |  | **Q1** | | | **Q2** | | | **Q3** | | | **Q4** | | |
|  | **ORCHID Range** |  | [-13.5;103] | | | | [-13.5;41.5[ | | | [41.5;52[ | | | [52;64[ | | | [64;103[ | | |
|  | **n=** |  | 293 | | | | 77 | | | 58 | | | 68 | | | 90 | | |
| **Nutrient intake and diet quality measures** | **ANOVA p-value sex** | **Absolute difference in means between men and woman (%)** | **Mean** | **SD** | **Pearson Correlation** | **Pearson 𝝆-value** | **Mean** | **95% CI** | | **Mean** | **95% CI** | | **Mean** | **95% CI** | | **Mean** | **95% CI** | |
| Energy intake (kcal/day) | < 0.001 | 26 | 2098.4 | 691.9 | 0.03 | 0.56 | 2065.6 | 1800.5, | 2330.8 | 1983.3 | 1753.1, | 2213.5 | 2183.7 | 1893.1, | 2474.2 | 2155.8 | 2008.9, | 2302.8 |
| Fibers (g/day) | < 0.001 | 17.3 | 21.9 | 7.8 | 0.32 | < 0.001 | 18.4 | 16.0, | 20.7 | 19.9 | 17.8, | 22.0 | 23.7 | 20.5, | 26.9 | 25.5 | 23.7, | 27.2 |
| Sugars (g/day) | < 0.001 | 16.1 | 89.7 | 41.2 | 0.07 | 0.37 | 87.8 | 70.0, | 105.5 | 87.7 | 75.9, | 99.6 | 83.4 | 71.8, | 95.0 | 99.8 | 91.8, | 107.8 |
| Saturated fat (% energy) | < 0.001 | 21.3 | 32.8 | 14.0 | -0.04 | 0.35 | 33.9 | 28.4, | 39.4 | 29.8 | 24.8, | 34.7 | 34.4 | 30.1, | 38.6 | 33.1 | 29.5, | 36.7 |
| Sodium (mg/day) | < 0.001 | 23.5 | 3576.4 | 1475.1 | 0.05 | 0.29 | 3346.8 | 2879.3, | 3814.2 | 3230.8 | 2764.6, | 3697.1 | 3955.7 | 3324.7, | 4586.7 | 3759.3 | 3415.6, | 4102.9 |
| SED (kcal/100g) | < 0.001 | 9.5 | 148.0 | 33.9 | -0.31 | < 0.001 | 166.6 | 154.2, | 179.1 | 150.3 | 137.6, | 163.1 | 141.6 | 134.2, | 149.0 | 133.0 | 124.3, | 141.6 |
| Moderation subscore | < 0.001 | -10.7 | 59.1 | 11.7 | 0.003 | 0.86 | 59.8 | 54.9, | 64.8 | 60.0 | 55.0, | 65.0 | 58.3 | 55.2, | 61.4 | 58.2 | 55.5, | 60.9 |
| MAR (% adequacy) | 0.001 | 4.2 | 86.4 | 9.7 | 0.31 | < 0.001 | 82.5 | 77.9, | 87.0 | 83.9 | 80.0, | 87.7 | 88.8 | 86.1, | 91.4 | 90.6 | 89.3, | 91.8 |
| Adequacy subscore | 0.002 | 8.6 | 69.0 | 15.2 | 0.33 | < 0.001 | 63.4 | 57.0, | 69.8 | 64.4 | 58.6, | 70.3 | 72.5 | 67.2, | 77.7 | 75.8 | 73.4, | 78.1 |
| Total intakes (g/day) | 0.003 | 10.3 | 2832.6 | 862.9 | 0.16 | 0.006 | 2718.9 | 2419.1, | 3018.8 | 2753.4 | 2466.3, | 3040.4 | 2870.3 | 2548.6, | 3192.0 | 2987.5 | 2784.4, | 3190.5 |
| Proteins (g/Kg/day) | 0.69 | 1.08 | 1.1 | 0.4 | 0.20 | < 0.001 | 1.0 | 0.9, | 1.1 | 1.1 | 0.9, | 1.2 | 1.1 | 1.1, | 1.2 | 1.2 | 1.2, | 1.3 |
| PANDiet | 0.21 | 1.2 | 64.1 | 5.7 | 0.43 | < 0.001 | 61.6 | 59.9, | 63.3 | 62.2 | 60.6, | 63.9 | 65.4 | 63.5, | 67.2 | 67.0 | 65.8, | 68.1 |

Supplemental table 4 continued: Relations between nutrient intake, diet quality measures and ORCHID by sex

|  | **Women** | | | | **ORCHID quartiles among women (n= 403 )** | | | | | | | | | | | |
| --- | --- | --- | --- | --- | --- | --- | --- | --- | --- | --- | --- | --- | --- | --- | --- | --- |
|  |  |  |  |  | **Q1** | | | **Q2** | | | **Q3** | | | **Q4** | | |
|  | **ORCHID Range** | [-36;115] | | | [-36;43.5[ | | | [43.5;53.5[ | | | [53.5;63.4[ | | | [63.4;115] | | |
|  | **n=** | 403 | | | 94 | | | 99 | | | 96 | | | 114 | | |
| **Nutrient intake and diet quality measures** | **Mean** | **SD** | **Pearson Correlation** | **Pearson 𝝆-value** | **Mean** | **95% CI** | | **Mean** | **95% CI** | | **Mean** | **95% CI** | | **Mean** | **95% CI** | |
| PANDiet | 64.86 | 6.36 | 0.44 | < 0.001 | 60.69* | 59.3 | 62.1 | 65.15 | 63.8, | 66.5 | 66.10 | 64.7, | 67.5 | 67.55 | 66.3, | 68.8 |
| Fibers (g/day) | 18.65 | 6.46 | 0.36 | < 0.001 | 15.97 | 14.0 | 18.0 | 18.42 | 17.0, | 19.8 | 18.51 | 16.5, | 20.5 | 21.75 | 20.2, | 23.3 |
| Adequacy subscore | 63.59 | 16.52 | 0.31 | < 0.001 | 54.65* | 49.4 | 59.9 | 65.11 | 61.8, | 68.4 | 64.14 | 59.8, | 68.5 | 70.60 | 67.1, | 74.1 |
| MAR (% adequacy) | 82.93 | 11.40 | 0.30 | < 0.001 | 76.26* | 72.1 | 80.4 | 84.20 | 82.1, | 86.3 | 83.81 | 81.1, | 86.5 | 87.55 | 85.4, | 89.7 |
| Total intakes (g/day) | 2567.87 | 732.06 | 0.21 | < 0.001 | 2718.95 | 2100.0 | 2498.5 | 2753.36 | 2351.0, | 2711.9 | 2870.28 | 2458.9, | 2840.3 | 2987.47 | 2632.6, | 2954.0 |
| Sodium (mg/day) | 2895.63 | 1275.55 | 0.10 | 0.09 | 2649.49 | 2241.4 | 3057.6 | 3003.13 | 2617.3, | 3389.0 | 2954.14 | 2572.3, | 3336.0 | 2977.76 | 2708.3, | 3247.2 |
| Proteins (g/Kg/day) | 1.10 | 0.42 | 0.04 | 0.35 | 1.05 | 0.9 | 1.2 | 1.14 | 1.0, | 1.3 | 1.10 | 1.0, | 1.2 | 1.11 | 1.0, | 1.2 |
| Moderation subscore | 66.14 | 10.99 | 0.02 | 0.67 | 66.73 | 63.2 | 70.3 | 65.19 | 62.7, | 67.6 | 68.06 | 64.7, | 71.5 | 64.50 | 61.7, | 67.3 |
| Energy intake (kcal/day) | 1665.06 | 551.41 | 0.02 | 0.74 | 1563.12 | 1355.3 | 1770.9 | 1779.60 | 1640.5, | 1918.7 | 1602.37 | 1447.6, | 1757.1 | 1718.89 | 1615.9, | 1821.9 |
| Sugars (g/day) | 77.24 | 32.02 | -0.01 | 0.84 | 70.57 | 59.9 | 81.2 | 79.23 | 71.5, | 86.9 | 74.13 | 67.2, | 81.0 | 85.25 | 78.1, | 92.4 |
| Saturated fat (% energy) | 27.06 | 12.88 | -0.07 | 0.31 | 26.05 | 22.0 | 30.1 | 30.25 | 26.6, | 33.9 | 25.20 | 21.2, | 29.2 | 26.81 | 24.5, | 29.1 |
| SED (kcal/100g) | 135.09 | 32.60 | -0.43 | < 0.001 | 146.46 | 136.1 | 156.8 | 145.50 | 137.2, | 153.8 | 124.99 | 116.9, | 133.1 | 123.53 | 117.4, | 129.7 |
| CI = confidence interval | | | | | | |  |  |  |  |  |  |  |  |  |  |
